# Electrochemical Sensors and Biosensors for the Analysis of Tea Components: A Bibliometric Review

**DOI:** 10.3389/fchem.2021.818461

**Published:** 2022-01-14

**Authors:** Jinhua Shao, Chao Wang, Yiling Shen, Jinlei Shi, Dongqing Ding

**Affiliations:** School of Chemistry and Bioengineering, Hunan University of Science and Engineering, Yongzhou, China

**Keywords:** electrochemical sensor, tea, antioxidant, caffeic acid, gallic acid, tea polyphenols, analytical chemistry

## Abstract

Tea is a popular beverage all around the world. Tea composition, quality monitoring, and tea identification have all been the subject of extensive research due to concerns about the nutritional value and safety of tea intake. In the last 2 decades, research into tea employing electrochemical biosensing technologies has received a lot of interest. Despite the fact that electrochemical biosensing is not yet the most widely utilized approach for tea analysis, it has emerged as a promising technology due to its high sensitivity, speed, and low cost. Through bibliometric analysis, we give a systematic survey of the literature on electrochemical analysis of tea from 1994 to 2021 in this study. Electrochemical analysis in the study of tea can be split into three distinct stages, according to the bibliometric analysis. After chromatographic separation of materials, electrochemical techniques were initially used only as a detection tool. Many key components of tea, including as tea polyphenols, gallic acid, caffeic acid, and others, have electrochemical activity, and their electrochemical behavior is being investigated. High-performance electrochemical sensors have steadily become a hot research issue as materials science, particularly nanomaterials, and has progressed. This review not only highlights these processes, but also analyzes and contrasts the relevant literature. This evaluation also provides future views in this area based on the bibliometric findings.

## Introduction

Tea is one of the most popular natural health drinks and is deeply ingrained in people’s lives. Tea trees are grown in around 30 nations throughout the world, yielding roughly 2.5 million tons of tea every year. The introduction of tea trees, on the other hand, can be easily influenced by soil and climate conditions (Feng et al., 2010; Wambu et al., 2017; Kottawa-Arachchi et al., 2019; Beringer et al., 2020). Furthermore, the nutritional composition and taste of tea fluctuate significantly due to variances in processing processes. Tea leaves are classified in a variety of ways based on various factors (Liu et al., 2015). Green tea, yellow tea, white tea, oolong tea, black tea, and dark tea are the most often used criterion for categorizing tea based on the degree of fermentation (de Carvalho Couto et al., 2021). Green tea is produced without the use of fermentation. Yellow tea is fermented to a degree of 10–20 percent. White tea is fermented to a percentage of 10–30%. Oolong tea is fermented to a degree of 20–60%. Black tea is fermented to a degree of 80–90 percent. Dark tea is a post-fermented tea, meaning it has undergone the most fermentation. The material components in tea leaves are changed into diverse forms as a result of varying degrees of fermentation (Zheng et al., 2016; Marx et al., 2017; Seth et al., 2019). Unfermented green tea, for example, preserves more of the natural components of the fresh leaves. These nutrients offer unique therapeutic properties in the human body, including anti-aging, anti-cancer, anti-inflammatory, and antiseptic properties (Kochman et al., 2021). Oolong tea processing, on the other hand, employs alternating mechanical force and stacking (Ng et al., 2018). The external force damages the cellular tissue of the leaf edge, while the polyphenols are oxidized and undergo other chemical changes as a result of the mechanical activity.

**FIGURE 1:**
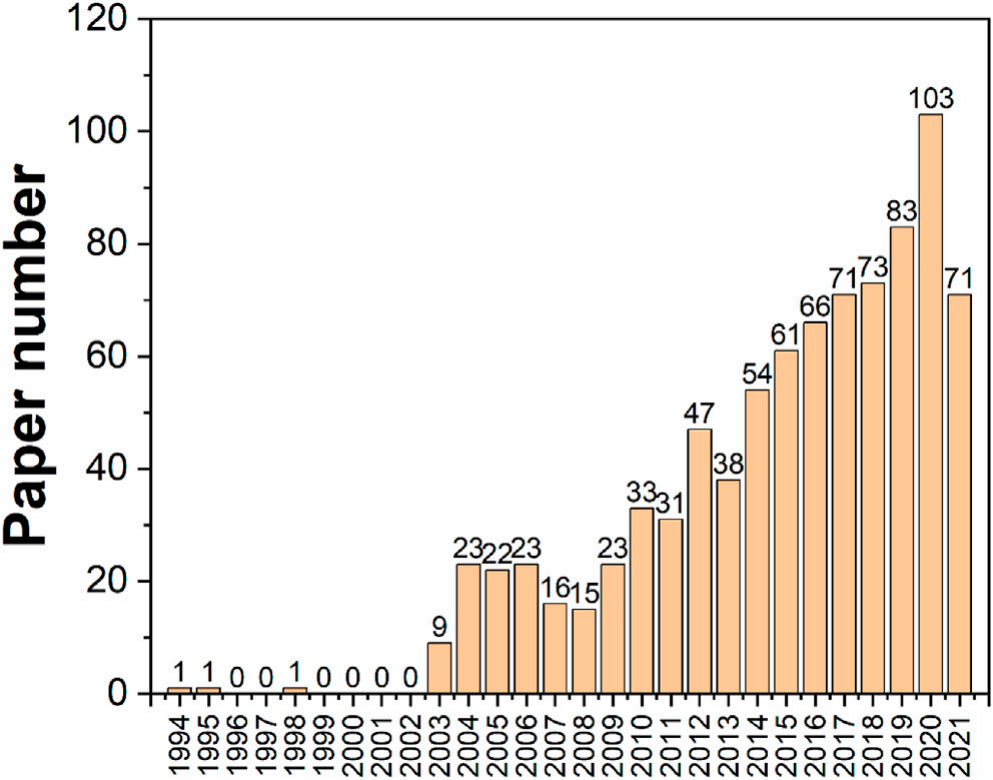
Statistical histogram of research on tea using electrochemistry from 1994 to 2021.

Tea leaves contain about 700 chemicals that have been extracted and identified. The secondary metabolic components of tea, such as tea polyphenols, amino acids, alkaloids, aromatic chemicals, pigment molecules, and so on, are primarily responsible for its distinctive flavor (Chapagain and Hoekstra, 2007; McCants, 2008; Tanui et al., 2012; Mzembe et al., 2016; Wang et al., 2020; Ying et al., 2020; Zhang et al., 2020; Zhou et al., 2020). They have a close association with specific pharmacological effects and impact the quality and flavor of tea (Koch et al., 2018; Wei et al., 2018). Based on the characteristics of the components, tea component analysis may be separated into two primary types: flavor component and quality component (Xu et al., 2019a). The flavor component is linked to the color, aroma, and taste of tea, and its detection focuses on determining the flavor qualities of tea quality (Xu et al., 2019b). The detection of the quality component is primarily for quality control and inspection purposes (Wang et al., 2019). The most often utilized techniques are colorimetric and spectroscopic approaches (Zhi et al., 2017). However, in the recent decade, the rapid development of electrochemical sensing techniques has enticed many scientists to experiment with electrochemical biosensing approaches to assess tea components. High sensitivity, wide linear response, outstanding stability, and reproducibility are all advantages of electrochemical sensors. Furthermore, the low cost of electrochemical measurements is a significant advantage. Electrochemical sensors consist of an electrochemical cell with at least two electrodes to form a closed electrical circuit and a transducer where charge transport (always electronic) takes place, whereas charge transport in the analyte sample can be electronic, ionic, or mixed (Power et al., 2018; Karimi-Maleh et al., 2021a). Electroanalytical methods are a class of analytical chemistry techniques that measure the potential (volts) and/or current (amperes) in an electrochemical cell containing the analyte to investigate it (Naveen et al., 2017; Karimi-Maleh et al., 2021b; Karimi-Maleh et al., 2021c; Karimi-Maleh et al., 2021d; Mani et al., 2021; Karimi-Maleh et al., 2022). Two important electrochemical methods used in the parameter evaluation of tea products are voltammetry, which measures current as potential varies, and electronic sensing, which includes the electronic nose (E-nose), electronic tongue (E-tongue), and electronic eye (E-eye). ASV, CV, SWV, and staircase voltammetry, in particular, use various types of electrodes, such as inert carbon electrodes, glassy carbon electrodes, and paraffin-impregnated graphite electrodes, to help determine the quality of tea products. We searched the WOS core database using the keywords *electrochemistry* and *tea* and discovered a total of 865 papers dedicated to the study of tea using electrochemistry from 1994 to 2021, as shown in Figure 1. It’s worth noting that similar research has gotten a lot of attention in the recent decade.

**FIGURE 2:**
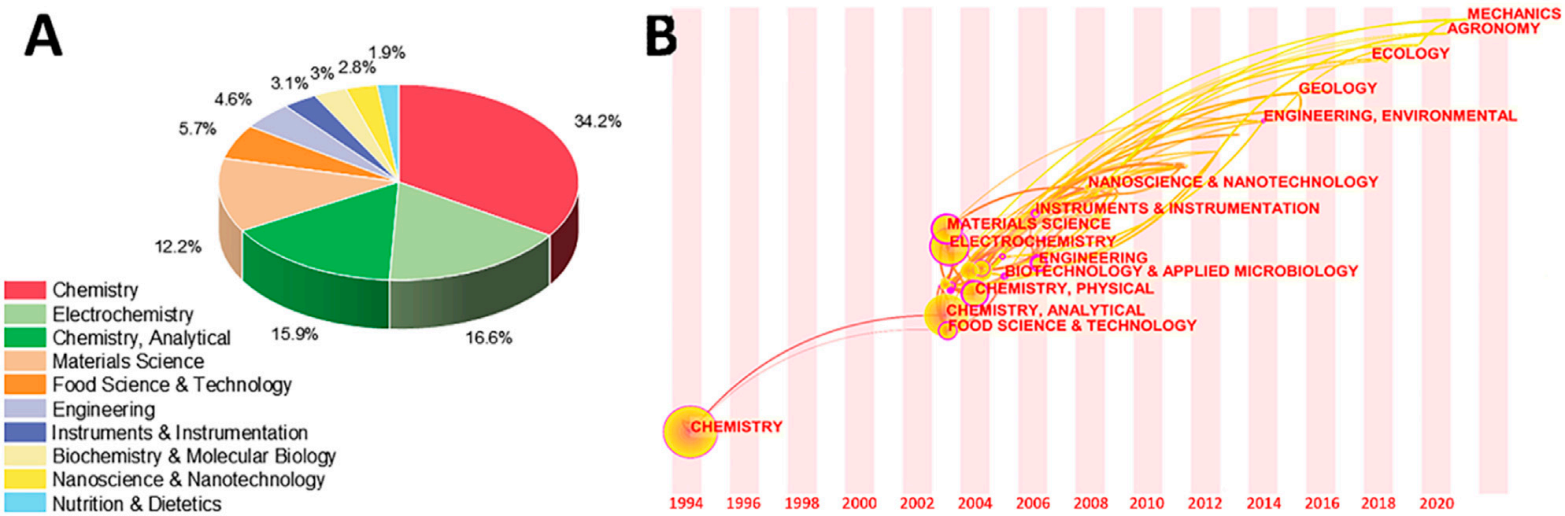
**(A)** The amount of contribution of electrochemical sensing techniques for tea research in different categories. **(B)** Variation of category with year in the literature of electrochemical sensing techniques for tea analysis.

We conducted a bibliometric analysis of these 865 studies to see if electrochemical biosensing may replace existing analytical approaches in tea quality control. For the bibliometric analysis and visual presentation, CiteSpace was employed (Chen, 2004; Chen, 2006; Chen and Song, 2019). The analysis report compares electrochemical techniques for tea analysis in terms of procedural and thematic changes. We also kept an eye on the cutting-edge of electrochemical sensing technologies in the field of tea analysis. We included both the analysis and data comparison of specific publications in traditional review writing, in addition to the overall pulse of bibliometric analysis. The use of a mix of electrochemical biosensing techniques and nanomaterial technologies was highlighted in particular. The most representative of these pieces was also thoroughly examined.

## Literature Information Analysis

### Changes in the Literature Category of Electrochemical Sensing Technology for Tea Analysis

Changes in electrical signals are the basis for signal production in electrochemical biosensing systems. Changes in current and resistance can be used as signals. Electrochemical oxidation-reduction is present in the bulk of these signal alterations. Under a result, the vast majority of tea research using electrochemical sensing technologies is classified as chemistry, electrochemistry, or analytical chemistry (Figure 2A). It’s important to note that each published paper does not fall into a single category. As a result, understanding other categories can assist in determining the target themes and areas to cross. What was unexpected, as indicated in Figure 2A, was the importance of materials science in this area. This is because the production of very sensitive electrochemical biosensors frequently necessitates the use of novel materials, according to a later study for the literature (Guth et al., 2009; Wang et al., 2015a; Alqarni et al., 2020; Ranga et al., 2021). This also helps to explain why instruments and instrumentation account for 4.6 percent of all category statistics. Similarly, nanotechnology and nanotechnologies accounted for 2.8 percent of the total. Traditional fields of tea research include food science and technology, biochemistry and molecular biology, and nutrition and dietetics. The development of electrochemical sensing technology has clearly been employed for tea research in various areas, as evidenced by this pie chart.

Figure 2B depicts a history of when these categories first appeared on this subject and how they interacted. The most significant categories arrived between 2003 and 2008, with the exception of chemistry, which debuted in 1994. This shows that the electrochemical sensing analysis approaches for tea research surge began in 2003, which is consistent with the results in Figure 1. The illustration also highlights the current multidisciplinary interaction of electrochemical analytical tools for tea research with ecology, geology, and agronomy. This indicates that the technology discussed in this article is well-established and has application potential, allowing it to be expanded into other areas for research. Abhradip and Chandan (Pal and Das, 2020a), for example, used electrochemical sensors to assess the solid tea waste extract’s ability to inhibit boiler quality steel under acidic circumstances.

### Author Country Distribution and Cooperation

**FIGURE 3:**
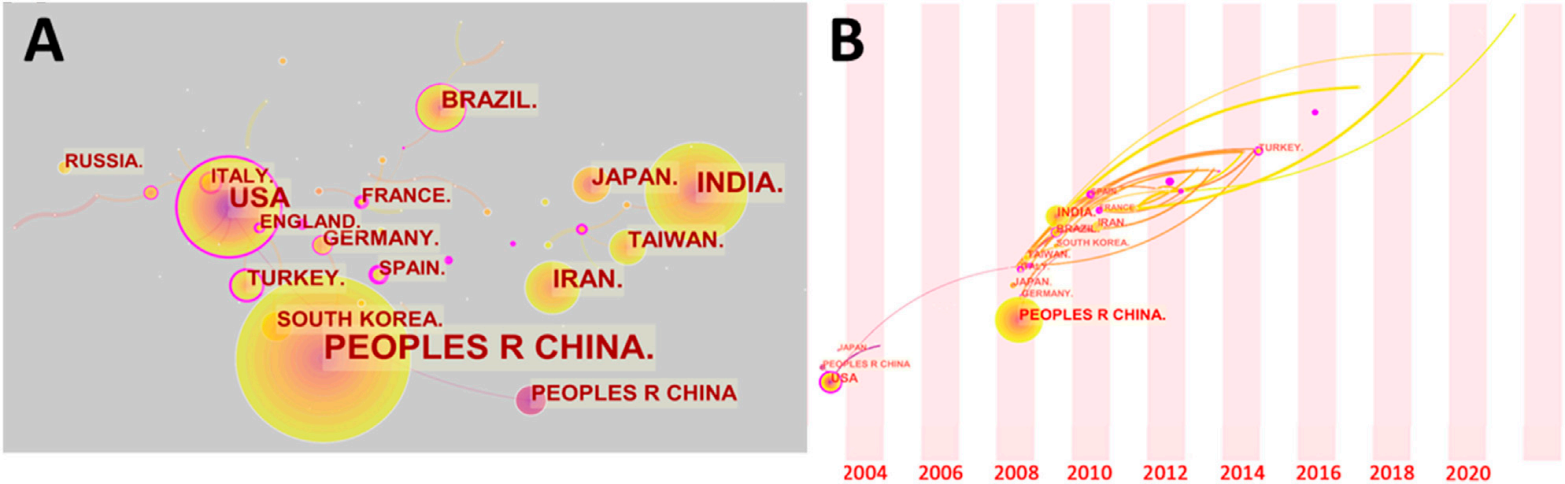
**(A)** Author’s country collaboration network map. **(B)** Time of involvement of different countries in electrochemical sensing research on tea.

Despite the fact that the tea tree is a widely produced plant, tea consumption in various countries has been influenced by cultural factors. As a result, in the scientific study of tea, there is relative independence between countries. Although many nations have actively investigated the development of electrochemical sensing technology in tea, as illustrated in Figure 3A, international cooperation is rare. Only a small percentage of articles have authors who are from different nations. The United States and the United Kingdom play a significant role in international cooperation on this issue. China and India, on the other hand, the two countries with the highest number of publications on this subject, have focused their research mostly on their own countries. Despite the fact that certain countries are geographically close, such as France and Germany, they continue to perform independent studies on this topic. Surprisingly, despite tea’s reputation as an Eastern beverage, the most influential publications in early studies were from the United States. The radical chemistry of epigallocatechin gallate and epigallocatechin was reported by Hagerman et al. (Hagerman et al., 2003). To validate the generation of hydroxyl radicals, the electrochemical redox potentials of both molecules were measured. For phenolic component identification, Luo et al. (Luo et al., 2003) employed liquid chromatography with coulometric electrochemical detection. In this investigation, tea blends were employed as a true sample. Figure 3B depicts the timelines of the various countries participated in this research. Many countries got actively involved in research on this topic between 2008 and 2010. Until recently, this topic continues to draw scholars from a variety of countries, who began to participate in the research. Cameroon, the Netherlands, and Finland, for example, have all published studies on this topic in the last 2 years. Dongmo et al. (Dongmo et al., 2020) from the University of Dschang developed an electrochemical biosensor for catechol detection in tea samples utilizing amino-grafting of montmorillonite. University of Yaoundé researchers Deutchoua et al. (Djitieu Deutchoua et al., 2019) devised two electrochemical techniques for determining antioxidant properties. As authentic samples, tea extracts were employed in this study. Overall, research on electrochemical sensing technology in tea is dominated by China, the United States, India, Brazil, Iran, and Japan. They were responsible for more than 70% of the academic papers.

**FIGURE 4:**
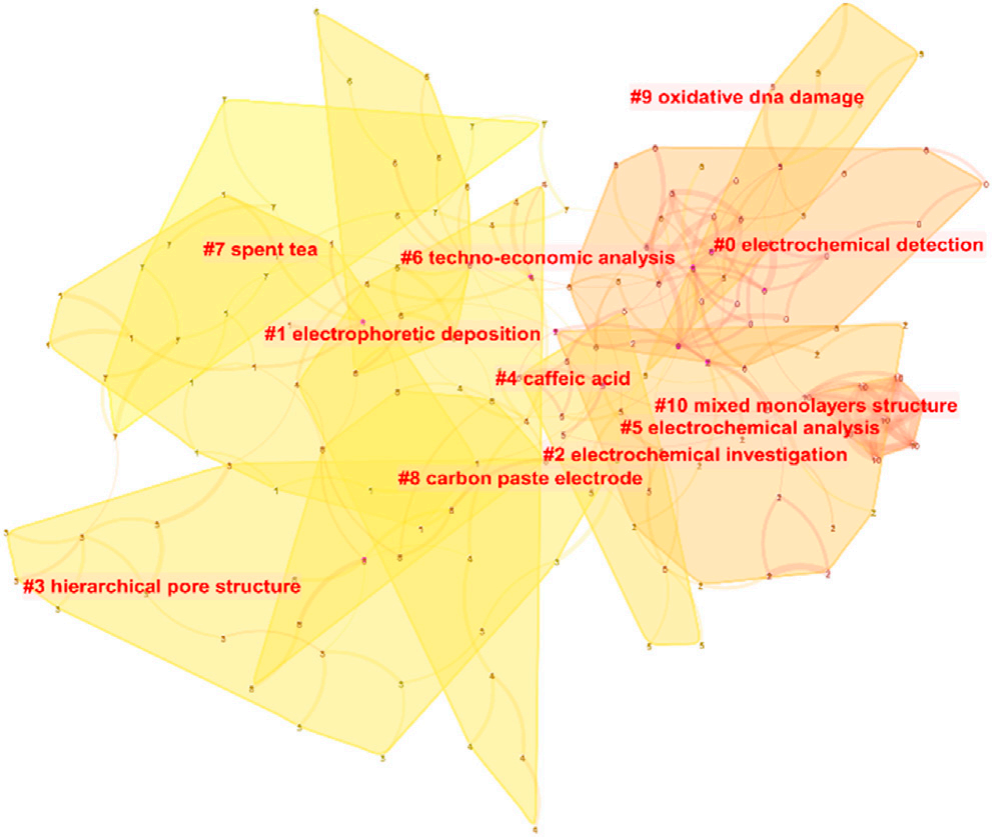
Cluster analysis of research content of electrochemical sensing for tea.

## Research Content Analysis

### Cluster Analysis of Research Content

Cluster analysis of the content reveals some of the most important study avenues for this subject. Electrochemical detection, electrophoretic deposition, electrochemical investigation, hierarchical pore structure, caffeic acid, electrochemical analysis, techo-economic analysis, spent tea, carbon paste electrode, oxidative DNA damage, and mixed monolayers structure were among the 11 top themes identified by bibliometric clustering of research on electrochemical sensing for tea (Figure 4). The results show that the information on electrochemical procedures, which includes analytical techniques and sensor preparation techniques, is the most important aspect of this topic. Malakootian et al. (Malakootian et al., 2020) used a carbon paste electrode modified with Eu^3+^-doped NiO to detect Pb (II) and Cd (II) in black tea.

**FIGURE 5:**
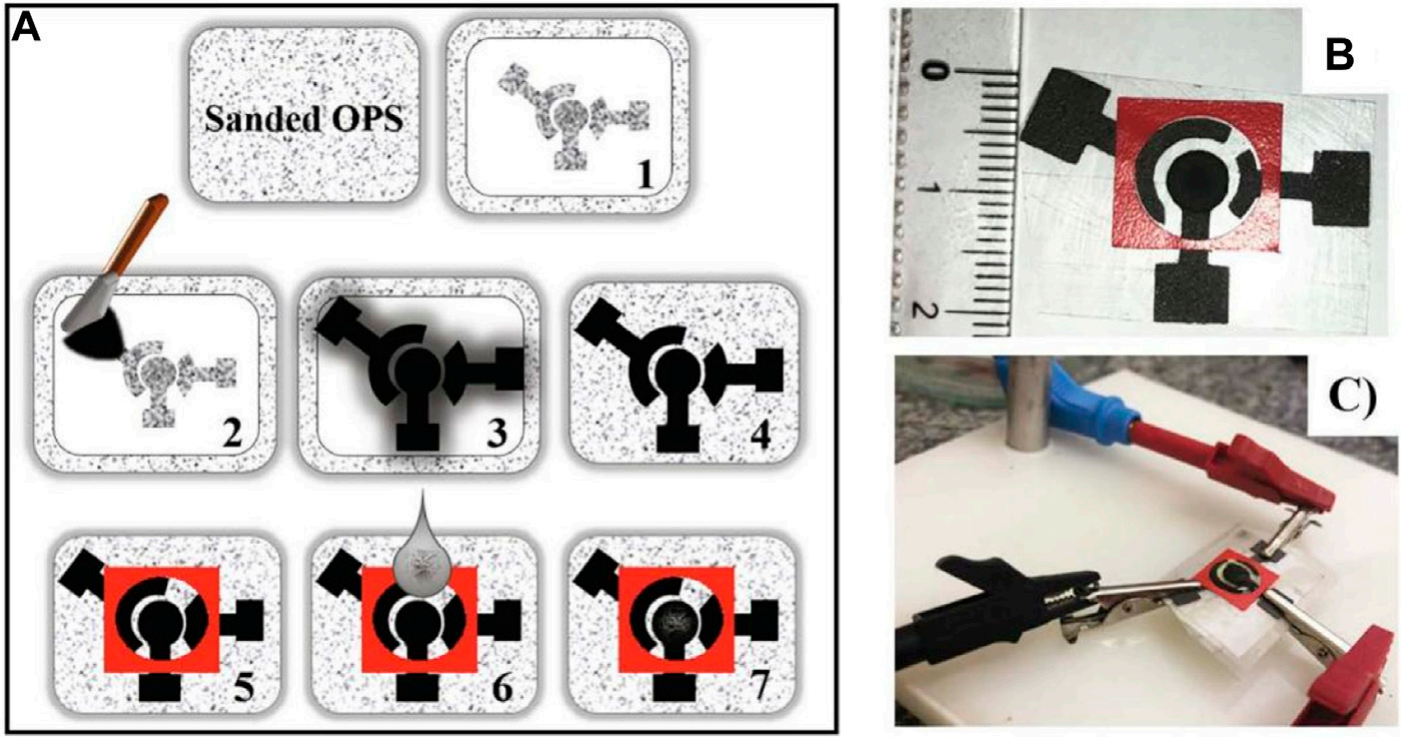
**(A)** Schematic representation, **(B,C)** images of SPE based caffeic acid biosensor (Araújo et al., 2020a). Copyright: Elsevier B.V.

The clustering analysis results included caffeic acid, which is particularly important in tea, in addition to the development of electrochemical techniques and sensors. For the detection of caffeic acid in tea leaves, many of these studies propose an electrochemical sensing device. Chang and colleagues, for example, suggested a ratiometric electrochemical sensor for the detection of caffeic acid (Yin et al., 2021). Caffeic acid is electro-oxidized with two electrons in a diffusion-controlled method. Caffeic acid’s hydroxyl groups undergo two-electron transfer and release two protons, resulting in the quick production of the matching quinone. The surface modification presented in this paper can help improve caffeic acid diffusion at the electrode. For caffeic acid detection, Arajo et al. (Araújo et al., 2020a) developed a screen-printed electrode modified with carbon nanotubes (Figure 5). However, limited research has been done to assess the antioxidant effects of caffeic acid in tea (Lima et al., 2020). Furthermore, because caffeic acid has substantial electrochemical activity, it has been utilized as a signal in various experiments to demonstrate successful caffeic acid production (Li et al., 2020).

Tea extracts are frequently utilized to examine the consequences of oxidative DNA damage due to their high antioxidant activity. Yury et al. (Kuzin et al., 2016), for example, described a GCE that had been electropolymerized with methylene blue. For analysis, the DNA solution was first combined with an oxidant before being immobilized on the modified GCE. By interrupting the DNA-methylene blue interactions, the voltammetric signal can be utilized to assess the degree of DNA damage (Kuzin et al., 2016). The presence of an antioxidant can help to slow down this process. They put this methodology to the test to see if it could determine the antioxidant capabilities of green tea extract. In addition to voltammetry, impedimetric technology (Kuzin et al., 2015). can be used for a similar purpose. Uliana and colleagues looked into whether tea may preserve DNA from dye-induced damage (Uliana et al., 2014). They proved that the tea solution could prevent adenine and guanine from reacting with the dye using electrochemical analysis. The current intensity of the adenine molecule had fallen by 60% of its initial value after an interaction duration of 180 s. Green tea appears to be able to minimize DNA molecule damage, according to the findings. Sumkova and Labuda (Šimková and Labuda, 2009) also suggested an electrochemical biosensor for detecting DNA damage and integrated the sensor into a commercial flow-through cell. This apparatus has been used to evaluate the antioxidative effects of ta extracts with great effectiveness.

Tea that has been discarded can be recycled as a valuable biological resource. High-temperature carbonization of wasted tea has been used to make electrodes or electrocatalysts in several investigations (Choi et al., 2016; Deng et al., 2016; Ahsan et al., 2020; Gao et al., 2021). In these investigations, electrochemical techniques were utilized as a characterisation tool to assess the performance of biochar. Gao et al. (Gao et al., 2021), for example, produced a biomorphic carbon electrode from discarded black tea and then used it to store potassium ions. Ahsan and colleagues constructed a cobalt-based electrocatalyst using discarded tea leaves as a template and then employed it for hydrogen and oxygen evolution and oxygen reduction (Ahsan et al., 2020).

### Keywords Analysis

**FIGURE 6:**
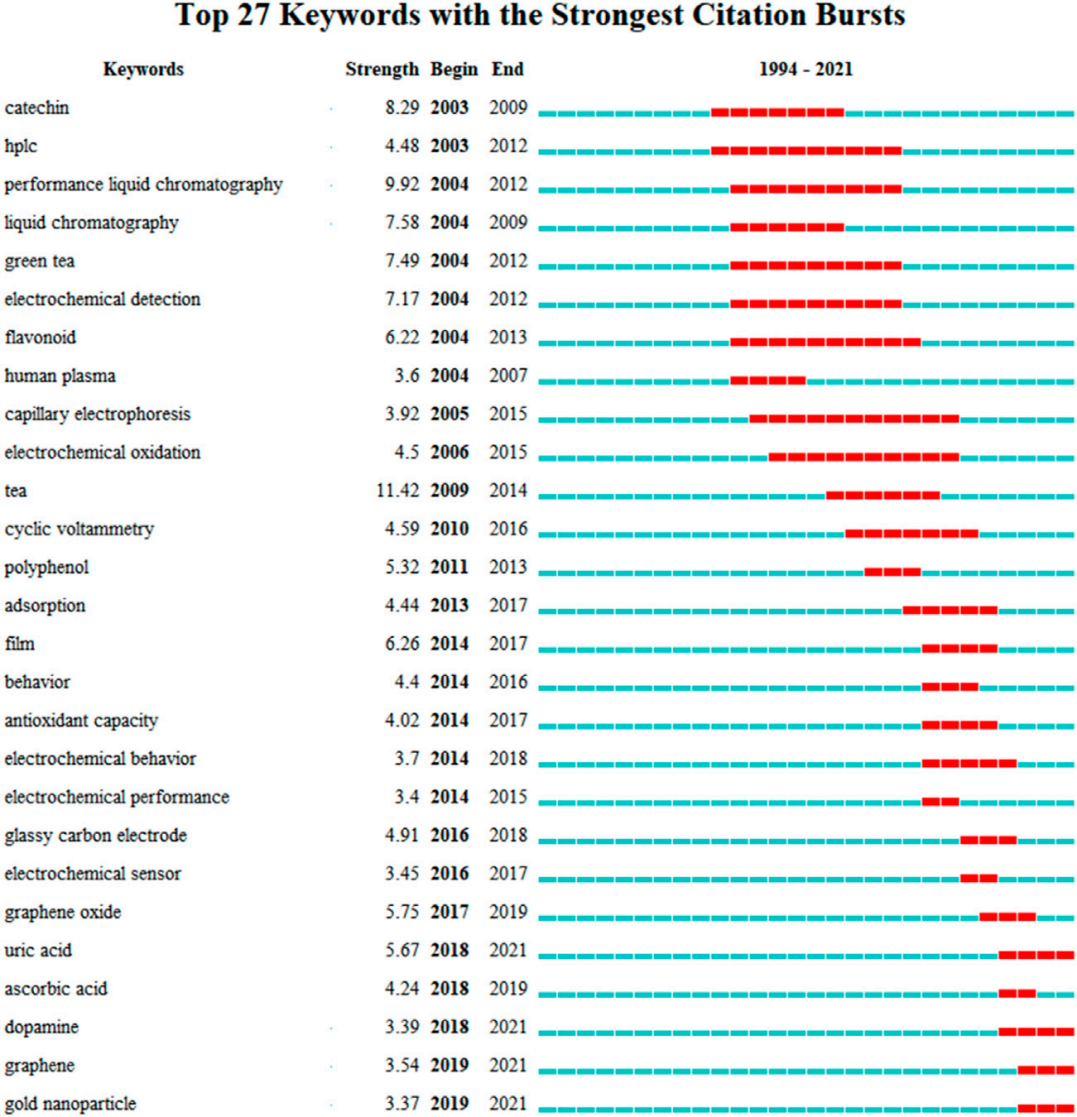
Top 27 keywords with the strongest bursts of electrochemical sensing techniques for tea analysis.

Keyword analysis can also reveal which research hotspots are being followed. The use of citation burst to analyze keywords might reveal how the study topic’s focus has shifted over time. The top 27 keywords with the greatest bursts of electrochemical sensing techniques for tea analysis are shown in Figure 6. The term bursts began in 2003, which was the year that additional research findings were published. Catechin, HPLC, performance liquid chromatography, green tea electrochemical detection, and flavonoid are some of the terms used in the beginning. The identification of catechin and flavonoids in tea using liquid chromatography is the main focus here. Electrochemical analysis was used as a technique for identifying samples after chromatographic separation of mixed samples at this point, rather than as a stand-alone sensing technique for tea detection. Long et al. (Hong et al., 2003), for example, suggested a method for detecting natural phenolic compounds in tea using liquid chromatography and multi-channel electrochemical detection. Similarly, Kotani et al. (Kotani et al., 2003) used an HPLC with electrochemical detection to identify catechins. Coulometric detection is frequently the most widely used electrochemical technology when paired with liquid chromatography (Chu et al., 2004; Kotani et al., 2007; Novak et al., 2010a; Shao et al., 2010; Narumi et al., 2014). Electrophoretic (Kartsova and Ganzha, 2006; Kartsova and Alekseeva, 2008) and voltammetric (Novak et al., 2010b; Hocker et al., 2017) approaches had previously been employed in conjunction with chromatographic methods to examine tea.

Around 2010, due to the development of electrochemical analysis techniques, a lot of work started to focus on the study of electrochemical behavior and antioxidant capacity measurement. It is worth noting that the studies of electrochemical behavior are not primarily an investigation of the electrochemical properties of tea components. According to the specific literature revealed, these works mainly focused on the use of extracts of tea leaves as inhibitors for the corrosion protection of metals. Changes in the electrochemical behavior of metals can be used as a characterization of the degree of corrosion. For example, Rauf et al. (Rauf and Mahdi, 2012) evaluated the effects of green tea on corrosion. Electrochemical frequency modulation and cyclic polarization scans were used for characterization. Unfortunately, the results showed that green tea did not show particularly excellent corrosion resistance. However, in a report by Tang et al. (Tang et al., 2018) green tea extract had good anti-corrosion efficacy on carbon steel. Pal and Das (Pal and Das, 2020b) also claimed that solid waste extract from tea factories is an excellent inhibitor.

The preparation of electrochemical sensors for tea detection has been the hottest topic since 2016. GCE has been chosen as the most commonly used commercial electrode. Different nanomaterials have been synthesized for the modification of GCE. Among them, graphene and its derivatives and gold nanoparticles have been studied the most.

### Electrochemical Biosensor Performance Comparison

According to the results of the preceding two sub-sections’ analyses, the most essential aspect of electrochemical biosensing in the research of tea is the detection of tea components. We used bibliometrics to summarize these specific works. The major characteristics (linear detection range, LDR, and limit of detection, LOD) of various electrochemical sensors for tea composition detection are summarized in Table 1.

**TABLE 1:** Comparison of the performance of electrochemical biosensors for the detection of different tea components.

Biosensor	Analyte	LDR	LOD	Year	Reference
Al/SiO_2_/CPE	Catechol	0.5–50 μM	0.1 μM	2009	Lin et al. (2009)
AgNPs/TiO_2_/ITO	Catechol	0.1–500 μM	0.05 μM	2012	Wang et al. (2012a)
CNT/carbon paper	Catechol	1–100 μM	0.29 μM	2013	Yue et al. (2013)
Ag doped TiO_2_/GCE	Catechol	1–15 μM	0.0249 μM	2016	Ravishankar et al. (2016)
Au@NG-PPy	Catechol	0.1–0.9 μM	0.0016 μM	2017	Vellaichamy et al. (2017)
Gr/GNRs/AgNPs/PPO	Catechol	2–2,300 μM	—	2018	Sandeep et al. (2018)
rGOSs@SrWO_4_	Catechol	0.034–672.64 μM	7.34 nM	2018	Manavalan et al. (2018)
Banana tissue/CPE	Catechol	1.4–15.7 mg/L	0.1 mg/L	2019	Broli et al. (2019)
Biomimetic oxidase/GO	Catechol	50–1,600 μM	0.09 μM	2021	Jiaojiao et al. (2021)
f-SWCNTs/PEDOTM/GCE	Catechin	0.039–40.84 μM	0.013 μM	2015	Yao et al. (2015)
Pt/MnO_2_/f-MWCNT/GCE	Catechin	2–950 μM	0.02 μM	2015	Ezhil Vilian et al. (2015)
(fMWCNT)/YHCF/GCE	Catechin	5–200 μM	0.28 μM	2015	Devadas and Chen, (2015)
N-doped carbon/GCE	Catechin	1–30 μM	0.088 μM	2017	Pang et al. (2017)
MIP	Catechin	5–100 μM	37 nM	2018	Chatterjee et al. (2018)
Cu@g-C_3_N_4_	Catechin	100–900 μM	15.12 μM	2021	Sanjay et al. (2021)
3DG/MWCNTs-Nc	Caffeic acid	0.2–174 μM	17.8 nM	2017	Sakthinathan et al. (2017)
Pt-PEDOT/rGO	Caffeic acid	5 nM–0.5 μM	2 nM	2018	Gao et al. (2018)
MWCNTs/SPE	Caffeic acid	2–50 μM	0.66 μM	2020	Araújo et al. (2020a)
MWCNT/SPEs	Caffeic acid	2–50 μM	0.2 μM	2020	Araújo et al. (2020b)
PMB@Ni–TPA/GCE	Caffeic acid	0.25–15.0 μM	0.2 μM	2021	Yin et al. (2021)
Poly-aspartic acid	Caffeine	0.25–30 μM	72 nM	2010	Wang et al. (2010)
Nafion/poly (safranine T)/GCE	Caffeine	0.3–100 μM	0.1 μM	2011	Guo et al. (2011)
MIPs/GNPs/MWNTs/GCE	Caffeine	0.5 nM-0.16 μM	90 pM	2012	Kan et al. (2012)
DNA-SWCNT/Nafion/GCE	Caffeine	0.02–1.5 μM	8 nM	2014	Wang et al. (2014)
PDDA-MWCNT	Caffeine	0.3–80 μM	0.05 μM	2017	Zhang et al. (2017a)
Polydopamine-gold	Caffeine	—	—	2017	Zhang et al. (2017b)
ZMWCNTMCPE/SDS/CPE	Caffeine	10–100 μM	75 nM	2019	Azab et al. (2019)
Nafion-NCNTs	Caffeine	0.08–6 μM	20 nM	2019	Wu et al. (2019)
SWCNT-SubPc	Caffeine	0.1–1.5 μM	13 nM	2019	Şenocak et al. (2019)
TiO_2_/MIP	Caffeine	5–120 μM	0.6 μM	2020	Das et al. (2020)
Cu-MOF/graphene	Caffeine	5–450 mM	1.38 mM	2021	Venkadesh et al. (2021)
Plasma-triggered polydimethylsiloxane/ITO	Caffeine	50 nM-700 μM	20 nM	2021	Li et al. (2021)
MoO_3_-GCNS	Caffeine	0.5–359 μM and 410–810 μM	21.24 nM	2021	Boopathy et al. (2021)
GC/Gr/SiC-NPs/[Cu(pydc) (apym)](2)	Caffeine	—	0.313 μM	2021	Hallaj et al. (2021)
Co_3_O_4_/GCE-Nafion	Caffeine	—	97 nM	2021	Kumar et al. (2021)
MIP(poly (o-phenylenediamine))	Epigallocatechin-3-gallate	0.5–10 μM	0.16 μM	2013	Duan et al. (2013)
MIP/GO/GC	Epigallocatechin-3-gallate	30 nM-10 μM	8.78 nM	2017	Liu et al. (2017)
Ni(OH)_2_ NPs	Epigallocatechin-3-gallate	10–100 mM	7 nM	2019	Nandy Chatterjee et al. (2019)
SWCNTs/poly-EB/GCE	Rutin	0.16–20 μM	82 nM	2012	Wang et al. (2012b)
SMWCNT-PEDOT-IL	Rutin	—	77 nM	2016	Nagles and García-Beltrán, (2016)
G-MWCNTs/GCE	Rutin	0.01–1 μM	5 nM	2016	Yang et al. (2016)
PEDOT/M-EDTA	Rutin	—	1.67 nM	2018	Lu et al. (2018)
NiCo2S4/rGO@PANI	Rutin	0.01–200 μM	0.007 μM	2018	Wang et al. (2018)
Polyphenol oxidase-AuNPs-mesoporous carbon	Rutin	1.6–28 mM	0.51 mM	2019	Zhong et al. (2019)
Poly (safranine/nano NiO)CPE	Rutin	16.1–230 nM	5.4 nM	2019	Saritha et al. (2019)
GQDs/PEDOT/GCE	Rutin	0.05–10 μM	11 nM	2019	Meng et al. (2019)
Fe_3_O_4_@TAPB-DMTP-COFs	Luteolin	0.01–70 μM	7.2 nM	2020	Xie et al. (2020)
MoO_3_-PPy NWs/MWCNTs	Luteolin	0.1 nM-10 μM	0.03 nM	2021	Zeng et al. (2021)
MIP	Morin	0.05–1.7 μM	0.01 μM	2016	Liu et al. (2016)
SiO_2_/CPE	Pyrogallol	2–300 μM	0.7 μM	2014	Tashkhourian and Ghaderizadeh, (2014)
PEI-rGO/GCE	Gallic acid	0.1–10 mg/L	0.07 mg/L	2013	Luo et al. (2013)
Polyepinephrine/GCE	Gallic acid	1–20 μM	0.663 μM	2013	Abdel-Hamid and Newair, (2013)
SPCE/PME	Gallic acid	—	0.076 μM	2015	Su and Cheng, (2015)
APTS@GO/PPAH-SDS/GCE	Gallic acid	0.006–2000 μM	1.7 nM	2018	Baghayeri et al. (2018)
PLM/MWCNT/GCE	Gallic acid	0.004–1.1 μM and 1.7–20 μM	3.1 nM	2019	Koçak et al. (2019)
Graphene/GCE	Gallic acid	80 nM–2 μM	1.2 nM	2019	Chen et al. (2019)
3D IPCNT/CNS/GCE	Gallic acid	0.05–20 μM	53 nM	2020	Zhao et al. (2020)
NG-Au@Ag NPs	Gallic acid	1–16.2 μM	3.17 nM	2020	Feng et al. (2020)
Silica gel/CPE	Quercetin	5–100 μg/L	3.53 μg/L	2012	Chen et al. (2012)
Porous alumina microfibers/CPE	Quercetin	0.025–1.5 μM	10 nM	2015	Li and Huang, (2015)
Platinum (II)-porphyrin/GCE	Quercetin	0.002–50 mg/L	0.8 μg/L	2015	Tian et al. (2015)
SWCNT/GCE	Quercetin	0.01–100 mM	7 mM	2019	Kuyumcu Savan, (2020)
GCE	Quercetin	7.9 nM-3.96 μM and 3.96–14.86 μM	2.2 nM	2020	Karaboduk and HASDEMİR, (2020)
Co_3_O_4_/GCE	Quercetin	0.01–3 mM	70 nM	2021	Khand et al. (2021)
MWCNTs-CS	Tea polyphenols	100–1,000 mg/L	10 mg/L	2009	Guo et al. (2009)
Diazonium-tyrosinase	Tea polyphenols	—	0.1 mM	2010	Cortina-Puig et al. (2010)
Pt NPs-rGO-laccase	Tea polyphenols	0.2–2 μM	2.75 μM	2013	Eremia et al. (2013)
Ferric chloride/GCE	Tea polyphenols	0.192–0.318 mg/L	—	2014	Chattopadhyay and Sarkar, (2014)
Iron phthalocyanine	Tea polyphenols	—	0.176 μM	2016	Maximino et al. (2016)
Chloramine-T/GCE	Tea polyphenols	—	0.674 mg/L	2016	Sen et al. (2015)
Tyrosinase- (Co-1.57 Al(OH) (_x_)SO_4_	Tea polyphenols	Up to 10 μg/ml	0.33 pg/ml	2017	Soussou et al. (2017)
Cassava fiber-iron nanoparticles/spE	Tea polyphenols	3.5–31.5 μM	0.1 μM	2021	Shi et al. (2021)
Cetyltrimethyl ammonium bromide/CPE	Theophylline	0.8–200 μM	0.185 μM	2009	Hegde et al. (2009)
CdSe/GCE	Theophylline	1.0–40 μM and 40–700 μM	0.4 μM	2012	Yin et al. (2012)
ED-GO/GCE	Theophylline	0.8–60 μM	0.01 μM	2013	Cui and Zhang, (2013)
SWCNT-LMC/Nafion/GCE	Theophylline	0.3–38 μM	0.08 μM	2013	Gao and Guo, (2013)
MWNT|MnO_2_/GCE	Theophylline	0.1–20 μM	0.01 μM	2015	Yang and Li, (2015)
WS_2_/AgNP/GCE	Theophylline	0.05–150 μM	3 nM	2015	Wang et al. (2015b)
AuNP/MWCNT/GCE	Theophylline	0.5–20 μM	90 nM	2018	da Silva et al. (2018)
AFW/Nf/GCE	Theophylline	0.1–160 μM	0.0028 μM	2019	Karthika et al. (2019)
MIP/SL-MoS_2_-BOMC/GCE	Theophylline	0.01–50 μM and 50–250 μM	5 nM	2019	Hu et al. (2019)
beta-NiS/Ppy	Theophylline	10 nM-900 μM	1 nM	2019	Muthukumaran et al. (2019)
DMN-AuNPs/GCE	Theophylline	0.05–2.0 μM	9.6 nM	2021	Zhang et al. (2021)
MoS_2_/MWCNTs	Carbendazim	0.04–100 μM	7.4 nM	2020	Zhu et al. (2020)
V_2_O_5_/G-C_3_N_4_/PVA/GCE	Folic acid	0.01–60 μM	1.74 nM	2020	Karthika et al. (2020)
Polyacrylamide (MIP)/graphite	Flavins	20–100 μM	14 μM	2017	Nandy Chatterjee et al. (2017)
MWNT/GCE	Tannins	0.4–200 μM	0.1 μM	2004	Lü, (2004)
3D-CS/rGO/GCE	Acetamiprid	0.1 pM-0.1 μM	71.2 fM	2020	Yi et al. (2020)
Ag/His-GQD/G	Acetamiprid	0.1 fM-5 pM	0.04 fM	2020	Dan et al. (2020)
SPE-Gr	Sibutramine	2–120 μM	0.3 μM	2019	Lima et al. (2019)
Diamond paste electrode	Pb (II)	10–100 pM	—	2004	(Raluca-Ioana Stefan (2004, 2004)
BioExt/MWCNTs/GCE	Cd (II)	0.05–5 μM	1.01 nM	2020	Incebay et al. (2020)
rGO/Sb/GCE	Pb (II); Cd (II)	0.1–3 μM; 0.1–3 μM	45.5 nM; 70 nM	2020	Nunes et al. (2020)
Eu^3+^ doped NiO/CPE	Pb (II); Cd (II)	0.8–165 μg/L; 0.8–165 μg/L	0.1 μg/L; 0.4 μg/L	2020	Malakootian et al. (2020)
Mn-TiO_2_ NTAs	Cd (II)	—	0.01 μM	2020	Jiang et al. (2020)

From Table 1, it can be seen that catechol, catechin, caffeine, rutin, gallic acid, quercetin, and tea polyphenols were the most detected tea components by electrochemical sensing technique. Acetamiprid, theophylline, Pb (II) and Cd (II) are the most frequently detected hazardous substances. This is due to the fact that excessive levels of pesticides and heavy metals in tea can lead to food safety problems. Overall, with the development of electrochemical sensing analysis technology, the detection range of different analytes has been enhanced while the detection limits have been reduced. For example, catechol had a detection limit of 0.1 M in the report in 2009 (Lin et al., 2009), which was reduced to 7.34 nM in 2018 (Manavalan et al., 2018). Among these, the widespread use of carbon nanomaterials has proven to be a game-changer. Due to the synergistic role of matrix or composite, carbonaceous materials (Graphene, CNTs, Carbon Nanofibres, and Mesoporous carbon, etc.) and conducting polymer materials act as reliable catalysts with metal oxide nanoparticles for the production of nonenzymatic sensors. It is worth noting that electrochemical sensors are a very sensitive detection technology. The sensors described above already meet the needs of detection, so the pursuit of high sensitivity does not necessarily have practical value. The focus of future electrochemical sensor research should be on how to improve stability and repeatability. Also, miniaturization of electrochemical sensors to fit field detection is an important direction.

## Key Authors and Papers Analysis

### Author Co-citation Analysis

**FIGURE 7:**
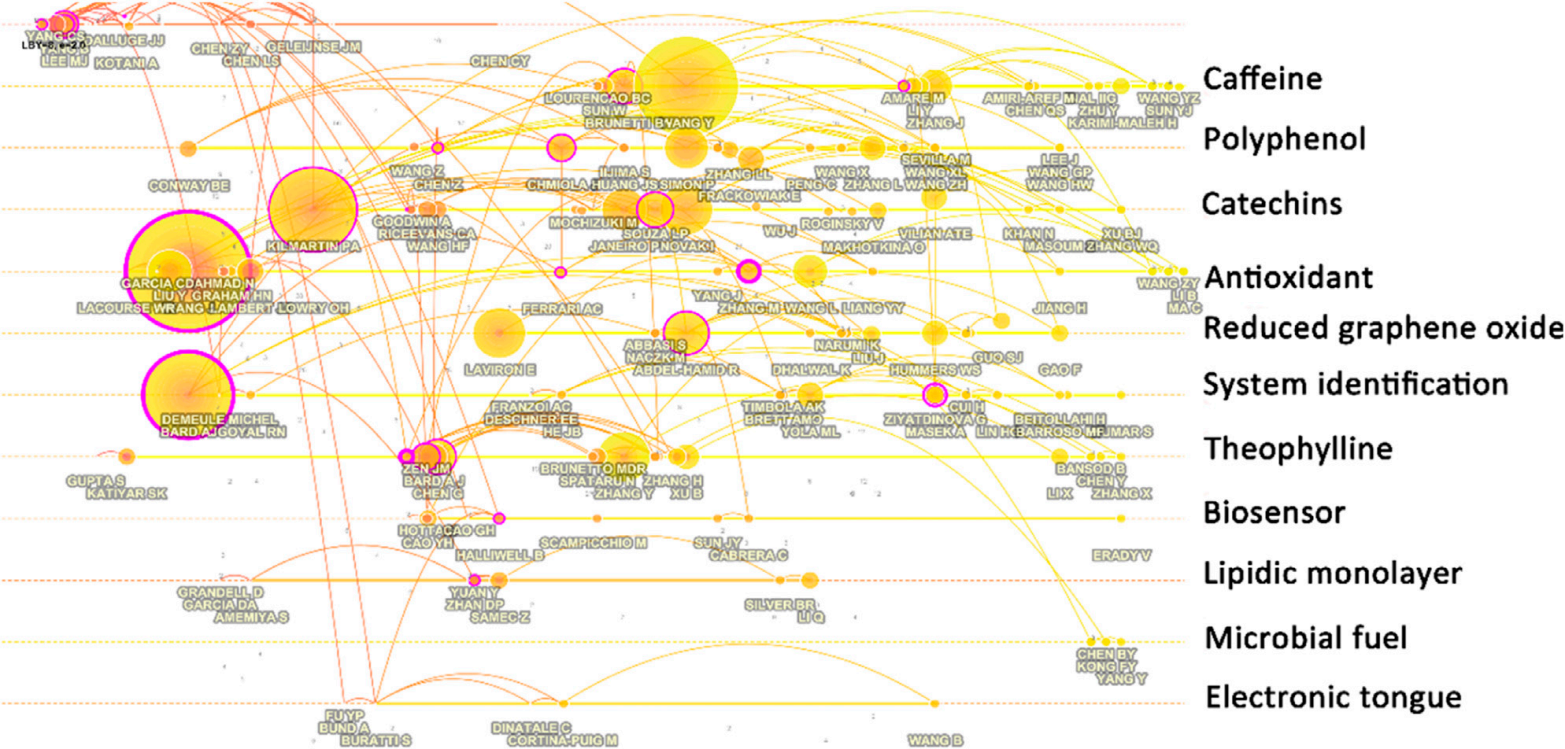
Author co-citation analysis with different research content clusters.

Figure 7 shows the relationship network of authors’ co-cited information. From this figure, it can be seen that the research work of those authors has had an impact on the field. It is worth noting that the work here is not necessarily limited to the study of electrochemical biosensing for tea, but rather exemplifies the type of work that has had a greater impact on the topic. Lee et al.'s (Lee et al., 2002) work on the pharmacokinetics of catechins and epigal-locatechin-3-gallate in human-consumed tea gave early insights into electrochemical studies on tea. Yang et al. (Yang et al., 2001) explored the antioxidant activity of catechins in microsomal lipid peroxidation at an early stage and also influenced the study of electrochemical techniques for tea detection. Studies on antioxidants in tea have also been mainly influenced by Kilmartin et al. (Kilmartin et al., 2001) because their work suggested for the first time that cyclic voltammetry is an excellent technique for the evaluation of antioxidant activity.

In the design and fabrication of electrochemical biosensors, the electrochemical methodology established by Bard and Faulkner (Bard and Faulkner, 2002) is the most important foundation. Wang’s textbook on analytical chemistry is also the basis for electrochemical sensor design (Wang, 2000). The study of the electron transfer process of glucose oxi-dase in glucose biosensors has laid the foundation for many subsequent mechanistic studies of biosensors (Liu et al., 2005). Meanwhile, the technique for rutin and quercetin detection in plants proposed by Chen et al. (Chen et al., 2000) was applied to the operation of a biosensor for tea analysis. Similarly, the technique for gallic acid detection proposed by Abdel-Hamid and Newair (Abdel-Hamid and Newair, 2013) also affects many of the later tests for substances in tea. The work conducted by Ziyatdinova et al. (Ziyatdinova et al., 2012) is also instructive for the detection of flavonoids. The discovery of graphene has become a very important material in the assembly of electrochemical sensors. The graphene-based bioenzyme sensors proposed by Yang et al. (Yang et al., 2011) have influenced the study of sensors targeting the detection of tea components. Electrochemical techniques are used in this work not only for the detection of analytes, but also as a method for the synthesis of nanomaterials.

### Reference Co-citation Analysis

**FIGURE 8:**
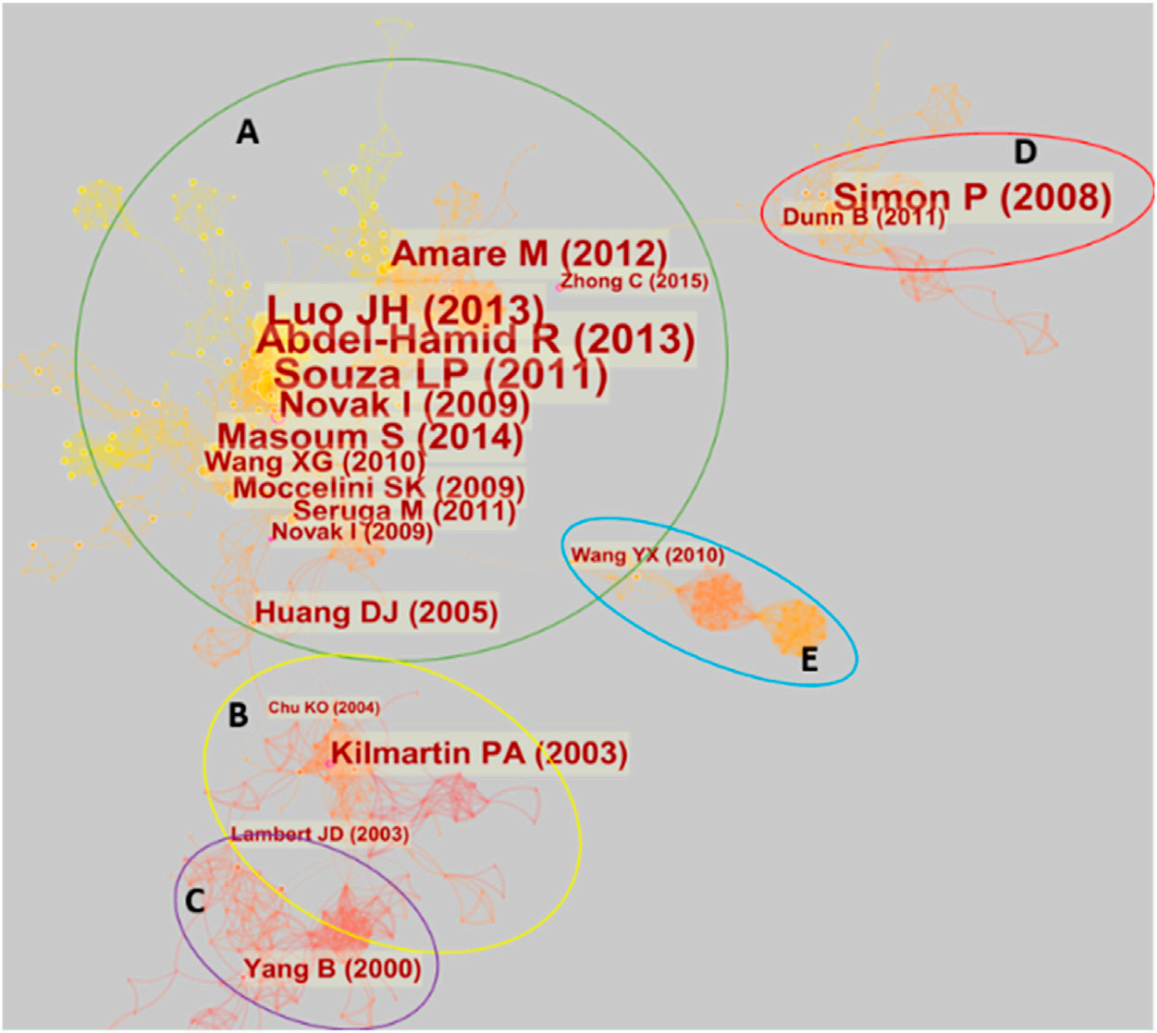
Reference co-citation analysis with five clusters.

Figure 8 illustrates the literature co-citation analysis relationship graph for electrochemical biosensors in tea analysis. As can be seen from the figure, the relationship between all the literature can be divided into five clusters, one of which contains a very large number of co-cited articles.

Cluster A has a dense network of pairs, representing work that is largely within a broad theme and is very closely linked. Important papers in this cluster include the work on gallic acid by Abdel-Hamind and Newair (Abdel-Hamid and Newair, 2013), previously mentioned in the authors’ co-citation analysis. Luo et al. (Luo et al., 2013) also proposed a method for gallic acid detection. Novak et al. (Novak et al., 2009) reported the electrochemical determination of epicatechin gallate using GCE. Masoum et al. (Masoum et al., 2014), Moccelini et al. (Moccelini et al., 2009) and Wang et al. (Wang et al., 2010) proposed electrochemical methods for catechin detection. Amare and Admassie (Amare and Admassie, 2012) proposed an electrochemical method for caffeine detection. Šeruga et al. (Šeruga et al., 2011) reported the method for polyphenols detection. All these techniques mentioned above are for the analysis of important components in tea.

Behind the detection of these substances is the passion of scientists for antioxidant substances. The explanation of the principle for antioxidant substances reported by Huang et al. (Huang et al., 2005) links cluster A and cluster B. In cluster B, the evaluation of the capacity of antioxidants using cyclic voltammetry reported by Kilmartin et al. (Kilmartin et al., 2001) is one of the most important works. A review of tea and tea polyphenols for cancer chemoprevention (Lambert and Yang, 2003) connects Cluster B and Cluster C. This links electrochemical sensing analysis to the specific health uses of tea components. Cluster C focuses on studies on the electrochemical behavior of tea components and human health, such as the relationship between the electrochemical oxidation of catechins and their antioxidant activity in microsomal lipid peroxidation (Yang et al., 2001).

Clusters D and E are two relatively independent groups. Cluster D focuses on tea as a source of carbon materials and has been used for energy storage. This cluster was accidentally included in the scope of this review because of the many electrochemical characterizations required in energy storage studies. This is a frequent occurrence in bibliometrics in sample statistics due to the sharing of similar keywords across different research directions. Cluster E is about the kinetic study of the electrochemistry of the tetraethylammonium/water interface. Since the abbreviation for tetraethylammonium is TEA, this literature was also accidentally included in the sample for this review.

## Conclusion and Perspectives

This bibliometrics-based review summarizes the progress of electrochemical analysis for tea component sensing. The following conclusions can be drawn based on the focus of research in different time periods:

1) Between 1994 and 2010, electrochemical techniques were often used as a detection step after the separation of samples by chromatographic techniques.
2) After the study of tea components gradually came to our attention, the electrochemical behavior of those components that have electrochemical activity began to be investigated.
3) Since some important components in tea have very pronounced electrochemical redox behavior, electro-chemical sensors are starting to become a technique to detect the concentration of these components.
4) As materials science, especially nanomaterials, has become a hot topic, the use of nanomaterials to improve the performance of electrochemical sensors has become the focus of this field. A large number of papers have been published from 2010 onwards.
5) Electrochemical techniques allow not only the detection of specific components in tea but also the evaluation of their antioxidant properties. Therefore, different methodologies based on electrochemical biosensing have been established for measuring the antioxidant properties of tea.

Based on the bibliometric survey of trends in this area, we believe that future direction is likely to focus on the following areas:

1) The use of novel nanomaterial composites, particularly carbon materials and noble metal nanoparticles, will continue to be popular in the design and fabrication of biosensors.
2) Antioxidant property detection biosensors based on DNA ligand technology may become the norm for evaluating tea’s antioxidant properties.
3) Because electrochemical sensors allow for rapid assessment of antioxidant properties, this technique can be applied to a wide range of *in vitro* biological experiments.
4) Miniaturization of electrochemical biosensors is an important step toward applying this technology in the field for food detection and quality control.
